# Targeting glycan sulfation in a CD11c+ myeloid population inhibits early KRAS-mutant lung neoplasia^1^^☆^^☆☆^

**DOI:** 10.1016/j.neo.2021.09.008

**Published:** 2021-10-26

**Authors:** So Young Kim, Scott C. Johns, Purva Gupta, Nissi Varki, Mark M Fuster

**Affiliations:** a Department of Medicine, Division of Pulmonary and Critical Care, University of California San Diego, La Jolla, CA, USA; b VA San Diego Healthcare System, Medical and Research Sections, La Jolla, CA, USA; c Glycobiology Research and Training Center, University of California San Diego, La Jolla, CA, USA; d Veterans Medical Research Foundation, San Diego, CA, USA

**Keywords:** Kras, tumor, Adenoma, Heparan sulfate, Cancer, Mutation, T cell, Macrophage, APCs, Antigen presenting cells, BSA, Bovine Serum Albumin, CD11c, CD11c locus, DC, Dendritic cell, Doxy, doxycycline, HS, Heparan sulfate, HSPG, Heparan sulfate proteoglycan, ICI, Immune checkpoint inhibition, IHC, Immunohistochemistry, LLC, Lewis lung carcinoma, MHC, Major histocompatibility complex, Ndst, N-deacetylase/N-sulfotransferase, TAM, Tumor associated macrophage, TTBS, Tween tris buffered saline, Treg, Regulatory T cell

## Abstract

Early lung carcinoma development may be modulated by innate host cellular mechanisms that promote tumor growth and invasion. We recently identified how a loss-of-function mutation in the glycan sulfating enzyme N-deacetylase/N-sulfotransferase-1 (Ndst1; involved in heparan sulfate biosynthesis) targeted to antigen presenting cells (APCs) may augment acquired anti-tumor T cell immune mechanisms. Crossing this mutation (*Ndst1*f/f *CD11cCre*+) onto a model of inducible spontaneous Kras mutant lung cancer [CCSP-rtTA; (tetO7) CMV-Kras-G12D] allowed us to examine how the APC mutation affects the formation and growth of early lung carcinoma. We examined early bronchocentric adenoma formation in the model, and the frequency of such events was significantly reduced on the mutant background. This was associated with significant reductions in tumor associated FOXP3+ cellular infiltration and CD163+ M2-type macrophage infiltration. The findings evolved prior to effector CD8+ T cell infiltration into tumors. The impact of this unique glycan under-sulfating mutation on inhibiting early Kras G12D mutant bronchocentric adenoma formation along with a cellular phenotype of inhibited tumor infiltration by cells involved in suppressive T-regulatory cell signaling (FOXP3+ cells) or tumor-permissive M2 macrophage functions (CD163+ cells) provides insight on how glycan targeting may modulate innate cellular mechanisms during early lung tumor development. The findings may also impact the future design of host-centered immunologic anti-tumor therapeutic strategies.

## Introduction

Early tumor formation may be subject to pressure or patterning by immune modulating cells. Targeting unique molecular modifications to immune cells in this transformation microenvironment may influence this process, and may be critical to identify and possibly harness in therapeutic designs. We recently published a body of experiments demonstrating that antigen presenting cells carrying a CD11c driven mutation in the major cell-surface glycan sulfating enzyme N-deacetylase/N-sulfotransferase (Ndst1) are associated with augmented induction of anti-tumor CD8+ cytotoxic T cells relative to that of wildtype antigen presenting cells (APCs) [1, 2]. The APC associated Ndst1 deficiency in such mutants probably exists predominantly in dendritic immune cells (DCs), and to a lesser extent in macrophages; with ex-vivo studies demonstrating that antigen presentation may be increased in such cells [1]. Altering the fine structure of surface glycans on such cells may also influence a balance of regulatory T cells, macrophages, and cytokines in lung tumors developing on this background, and this could further affect early tumor growth [3]. This may also precede induction of classical acquired immune mechanisms such as tumor antigen presentation or downstream T-cytotoxic pathways.

We applied these considerations to a Kras mutation driven lung cancer model that carries the above APC mutation leading specifically to under-sulfated chains of the glycosaminoglycan heparan sulfate (HS) on the APC surface. One of the hallmarks of altered immunity in early-stage lung cancer is the presence of regulatory/suppressor T cells (Tregs) within both tumor lesions as well as the blood. This is in addition to a program of intense immune suppression compared to that of normal surrounding lung [3]. Increased Treg cells have been found in mouse Kras-mutation models of lung carcinoma even prior to tumor formation, and their pre-tumor elimination markedly inhibits tumorigenesis [4]. In the bloodstream, T cells in the setting of early lung carcinoma also express immune-regulatory markers such as the checkpoint inhibitor PD1 on CD8+ effector T cells, presenting an early opportunity to destroy tumor cells that escape resection or ablative treatment as early metastases. Possibly, one might be able to “tip” a unique balance of Treg and effector T cells, or the level of APC immunosuppressive pathway expression through novel glycan-focused strategies that promote APC maturation [1, 2] or change APC effector functions that influence T cells prior to acquired immunity induction. This might provide an anti-tumor strategy independent of immune checkpoint inhibition (ICI); so that coupling the strategy with ICI in clinical/translational approaches may provide not only synergy but also efficacy in ICI insensitive states, including lung carcinomas with lower mutational burden.

In a recent study [1], we examined macroscopic subcutaneous Lewis lung carcinomas; and herein, we examine early events in spontaneous lung tumor formation in the unique glycan-targeted APC setting for the first time. The goal was not to examine carcinomatous tumor progression (with anti-tumor cellular immune effects under the *CD11cCre*+ driven *Ndst1* mutation, as in prior work), but to assess whether a mutation relegated only to APCs alters the earliest steps in spontaneous tumor formation in situ. It is curious that Treg cells seem to be important in possibly facilitating immune suppression early in tumor formation, while the roles of macrophages remain in question. This has been recognized in the Kras transgenic model lung tumor system [[4], [5], [6]]. Herein, we report early in tumor induction (at the stage of bronchocentric adenoma formation) on this unique genetic background, with effects of the myeloid-driven mutation on tumor formation and early cellular effectors.

## Materials And methods

We generated a model of Kras driven lung cancer, CCSP-rtTA; (tetO7) CMV-Kras-G12D, which is doxycycline inducible, in the laboratory; with kind provision of tetracycline operator-regulated responder (Kras G12D) and secretory protein - tet activator (CCSP-rtTA) mouse constructs [7] from the laboratory of Dr. J. Whitsett. In this system, upon doxycycline (doxy) administration, the CCSP-rtTA doxy-sensitive driver binds and drives the tetracycline responsive transgene (tetO7) CMV-Kras G12D expression. Mice did not carry tumor suppressor (e.g., P53) mutations. Mice were further crossed onto C57Bl/6 mice with a conditional mutation in *Ndst1* (*Ndst1*f/f) driven by the *CD11cCre* transgene, wherein *Ndst1* is inactivated in CD11c+ myeloid-derived APCs (predominantly classical DCs and macrophages). This results in under-sulfated HS on APCs of mutant tumor-bearing mice (designated as genotype CCSP+/KrasG12D+/ *Ndst1*f/f *CD11cCre*+; with *Cre*- littermates as controls). Lungs from mice during the period of 4 – 5.8 weeks following induction through doxycycline-supplemented chow (inducing early lung adenomatous tumor formation) were harvested and fixed in paraffin; and standard sections were processed under standard H&E staining. We quantified the density of adenomas per area of lung field in mutants and wildtype mice based on counts from each lung by a pathologist blinded to mouse genotype. Counts were taken as total nodules per lung (referenced to lung cross sectional area), and averaged from 2 levels from any given lung-block separated by 100μm microtome distance. Analysis was carried out for N=9 mutant (*Ndst1*f/f *CD11cCre*+) and N=7 wildtype (*Ndst1*f/f *CD11cCre*-) controls. Sub-analysis (also with blinding to genotype) involved scoring the frequency of dense adenomas that filled the airway lumen in mutant and wildtype mice.

For genotyping, PCR detection of the CCSP-rtTA transactivator transgene was detected using the following primers: fwd, 5′ –ACT GCC CAT TGC CCA AAC AC- 3′ and rev, 5′ –AAA ATC TTG CCA GCT TTC CCC- 3′. Detection of the (tetO7) CMV-Kras-G12D transgene was carried out using the primers:

fwd, 5′ –GGG AAT AAG TGT GAT TTG CCT- 3′ and rev, 5′ –GCC TGC GAC GGC GGC ATC TGC- 3′

Doxycycline was administered in the chow (200mg/kg) as a formulated standard chow diet (Teklan). Primers to detect the Ndst1 floxed (f/f) allele with reference to the *Ndst1*f/f *CD11cCre*+ mutation are as reported in [1].

Animal studies were approved by the local institutional animal-care-and-use-committee (IACUC).

Slides for antibody labeling were prepared by deparaffinization, re-hydration, and heated citrate antigen retrieval. Avidin/Biotin Blocking Kit (Vector) was used to minimize non-specific binding for biotinylated antibody applications. Anti-FOXP3 (Invitrogen) (1:100 dilution) labeling was carried out in 1% BSA/TTBS solution overnight at 4˚C. Biotin anti-rabbit secondary antibody (Vector) at 1:100 dilution in 1% BSA/TTBS solution was added to sections at room temperature for 1 hr. For CD8 staining, biotin-conjugated anti-Mouse CD8 antibody (Invitrogen) was used (1:100 dilution), with labeling carried out in 1% BSA/TTBS solution overnight at 4˚C. For tissue macrophage studies, unconjugated anti-mouse F4/80 antibody (Ebioscience) was used (1:100 dilution) for detection of tissue-differentiated macrophages, with biotin anti-rat antibody (Vector) at 1:100 dilution used as secondary; and anti-mouse CD163 antibody (Abcam) used (1:100 dilution) for detection of M2 subset macrophages, and biotin anti-rabbit antibody (Vector) at 1:100 dilution used as secondary. Alkaline Phosphatase Streptavidin and a Vector Blue Substrate Kit were used for detection (Vector). Counting of label positive cells in IHC stained tissue was carried out for all analyses blinded to genotype, and quantified as # cells per 40X high power field (HPF) quantified from either the lung background (averaged among 5 HPF fields per lung) irrespective of tumor, or as tumor-associated cell count analyses (tumor-associated cells/HPF, averaged from three tumor-associated fields, each taken from two independent levels through the tumor-bearing lung for any given mouse).

For statistics, the unpaired student's T-test was used for most analyses, while in one analysis the chi-square statistic was used. A value of *P* < 0.05 was used to determine statistical significance.

## Results

**Fig. 1:**
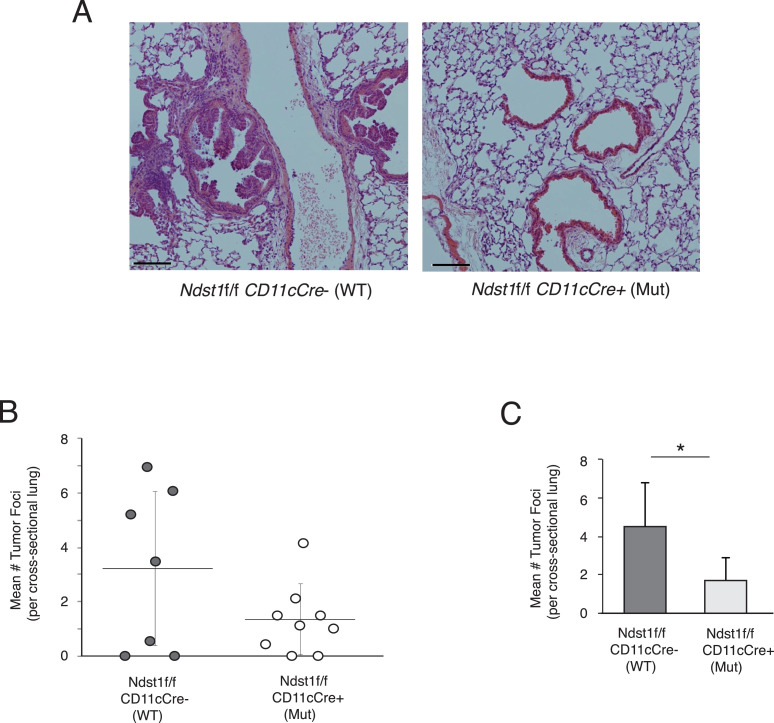
Under-sulfation of HS targeted to APCs results in decreased lung tumor formation in a spontaneous Kras lung carcinoma model. Spontaneous lung carcinoma was generated in a mouse model with inducible G12D transgenic mutation established in the laboratory. This was carried out on a compound mutant background wherein *Ndst1*f/f *CD11cCre*+ mutation (Mut) was crossed-in so that the effects of under-sulfated HS in *Cre*+ mutants (driven in *CD11c*+ APCs) on tumor growth could be compared to that of wildtype *Ndst1*f/f *CD11cCre*- (WT) controls. (A) Photomicrographs of H&E staining of the lung show representative lung sections from control (left) and mutant (right) mice, with two typical tumor foci (centered on airways/bronchi) shown in wildtype, while representative mutant lung section shows relatively normal bronchial epithelium (Bar=200μm). (B) Quantification of tumor foci, counting blinded to genotype in low-power fields on H&E sections shows counts presented on graph; with means (horizontal bars) and SD shown. (N=9 Mut mice and N=7 WT controls). (C) Among mice for which tumor foci could be detected by microscopy, the mean number of foci indexed to the hemi-thorax lung cross-sectional area is graphed (from lungs of N=7 Mut mice and N=5 WT controls); *P < 0.05 for difference in means. APC, antigen presenting cell; CD11c, CD11c locus; HS, Heparan sulfate;

*Spontaneous Kras-mutant lung carcinoma model demonstrates a lower frequency of early tumor foci in a setting of HS-altered APCs.* We generated an inducible model of Kras driven lung cancer, CCSP-rtTA; (tetO7) CMV-Kras-G12D [7] in the setting of mutation in a unique glycan sulfating biosynthetic enzyme. Mice were crossed onto a genetic background with a conditional mutation in *Ndst1* (*Ndst1*f/f conditional mutation mice) driven by the *CD11cCre* transgene expressed in myeloid-derived APCs (predominantly classical DCs and macrophages). At 4−5 weeks following doxycycline induction, adenomatous tumor development takes place, and we quantified the density of adenomas per area of lung field in mutants and wildtype mice (see representative photomicrographs in Fig. 1A) based on counts from each lung by a pathologist blinded to mouse genotype. Analysis of early lung tumors among all mice (N = 9 mutant and N = 7 wildtype controls) demonstrated a mean density of tumor foci in mutant lungs that was lower than that in wildtype lungs (Fig. 1B). Analysis of mouse lungs wherein any tumor was detected on microscopy analysis (from N = 7 mutant mice and N = 5 wildtype controls) revealed a significant reduction in the lung tumor density of mutant mice (Fig. 1C; *P* < 0.05 for difference in means). Moreover, *Ndst1*f/f *CD11Cre*+ mutants revealed a lower frequency of dense adenomas that filled the airway lumen relative to that of *Ndst1*f/f *CD11Cre*- wildtype littermates (observed on blinded pathologic review of 2/7 tumors in mutant lungs versus 4/5 scored in wildtype lungs; *P* < 0.01, chi-square goodness of fit statistic relative to wildtype).

**Fig. 2:**
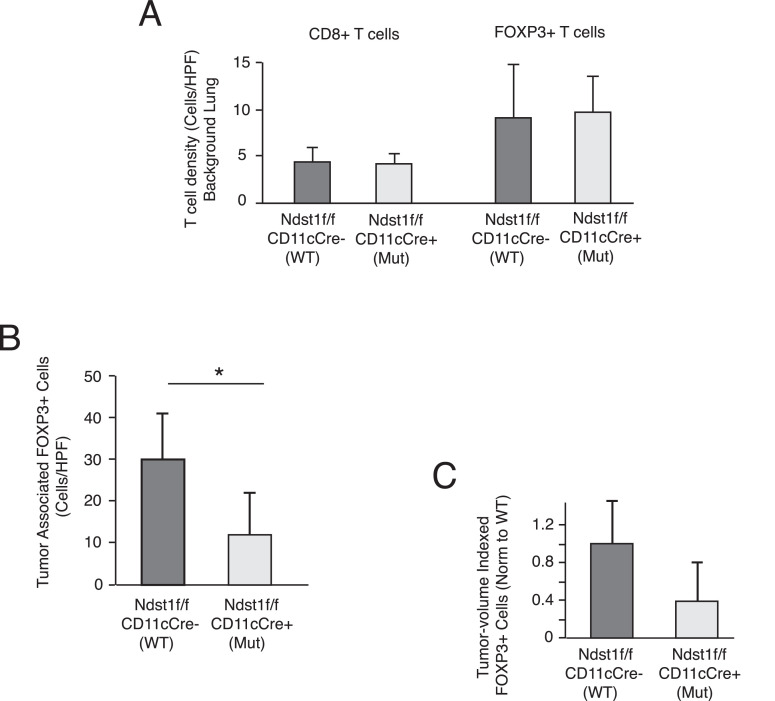
Lung tumor-associated FOXP3+ cell infiltration is reduced in *Ndst1*f/f *CD11cCre*+ mutant mice. (A) Initially, in IHC lung sections stained for CD8+ T cells and FOXP3+ cells, the background interstitial cell density (independent of tumor foci) for each cell type was quantified blinded to genotype (shown as mean # cells/HPF +/- SD; from n=4 mice/genotype). There were no significant differences between the respective cell densities for each genotype. (B) While CD8+ T cells did not show specific tumor-association in microscopic sections from the early Kras mutant tumors in lungs from either mutant or wildtype mice, FOXP3+ cells were found to infiltrate tumors, with mean tumor-associated cell densities (quantified blinded to genotype as cells/HPF; n=5 mice per group) in lung sections from *Ndst1*f/f *CD11cCre*+ mutant versus *Cre*- wildtype mice shown in the graph; *P < 0.05 for difference in means. Note mean density of tumor-associated FOXP3+ cells in lungs of WT mice as markedly elevated relative to that of background FOXP3+ cells shown in (A). The mean FOXP3+ cell density associated with lung tumors on the mutant genotype was also noted to be reduced relative to that of wildtype control mice when tumor-associated FOXP3+ cell densities were indexed to their respective mean tumor volumes (C); *P* = 0.08 for difference in mean values.

*Tumor-associated FOXP3+ cell infiltration is reduced in Ndst1f/f CD11cCre+ mutant mice.* A basal or background level of CD8+ and FOXP3+ cells (consistent with Treg cells) exists in the lung interstitium, with comparable densities (stained cells per HPF) for each cell type in mutant versus wildtype tumor-bearing lungs (Fig. 2A). A unique up-regulation of FOXP3+ cells was noted in association with the perimeter and body of tumors, while this was not the case for CD8+ T cells in such early tumors. (In fact, while CD8+ T cells had comparable densities (∼ 4.5 cells/HPF) in the background lung among genotypes, as in Fig. 2A, counting of specific tumor-associated CD8+ T cell densities (1.3/HPF for mutants and 3.1/HPF for wildtype) identified even lower densities than that of background lung.) Among Kras tumor-bearing wildtype mice, quantifying *tumor-associated* FOXP3+ cells in lung sections revealed a >3-fold induction of FOXP3+ cell density compared to that noted in the non-tumor background/interstitial lung tissue [compare mean FOXP3+ cell density in wildtype mice in Fig. 2A (∼ 9/HPF) to that noted in Fig. 2B (∼ 30/HPF)]. Interestingly, compared to that noted in lung tumors on the wildtype background, the mean level of tumor-associated FOXP3+ cells in mutant lungs was markedly reduced (Fig. 2B, and comparable to the background cell density in Fig. 2A). A similar reduction was noted upon indexing densities to the estimated mean tumor volume per nodule (to more strictly control for any effects of tumor size; *P* = 0.08 for difference in tumor volume indexed means in Fig. 2C): The reduction in FOXP3+ cell density indexed for tumor mass showed essentially the same trend, indicating what appears to be a distinct inhibitory effect of mutation on the unique tendency of FOXP3+ cells to associate or infiltrate the parenchyma of the early Kras tumors on the wildtype background.

**Fig. 3:**
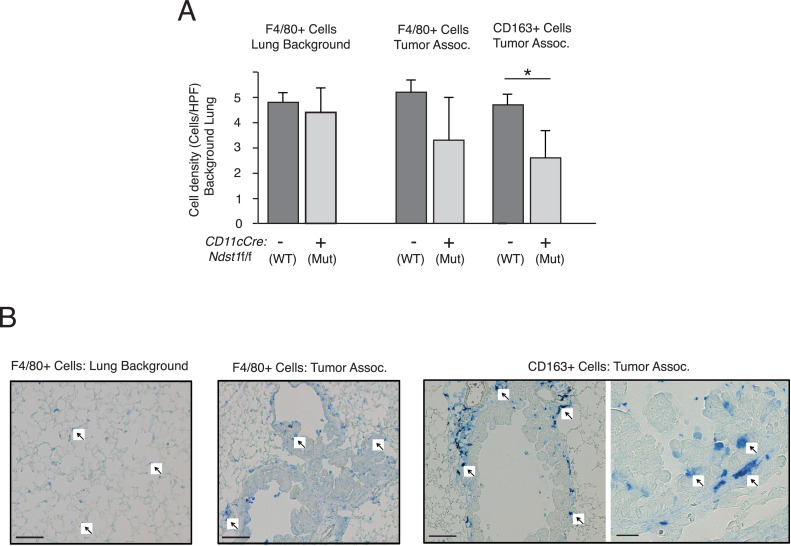
Lung tumor associated CD163+ cell infiltration is reduced in *Ndst1*f/f *CD11cCre*+ mutant mice. Macrophage IHC staining using F4/80 antibody as a general marker for tissue-differentiated macrophages in the lung as well as CD163 antibody to detect M2 subset tumor-associated macrophages was carried out on lung sections from Kras transgenic *Ndst1*f/f *CD11cCre*+ mutant mice and *Ndst1*f/f *CD11cCre*- littermates. IHC stain-positive cell densities (mean # cells/HPF +/- SD) were quantified blinded to genotype (from n=4 mice per genotype). (A) A diffuse pattern of F4/80 immunoreactivity throughout the lung showed a comparable cell density among genotypes (graph, left). Tumor-associated F4/80+ cell density was also not significantly different among genotypes (middle graph), albeit with a trend toward reduction in mutant tumors. On the other hand, CD163+ cells appeared to distinctly infiltrate tumors, and quantification revealed a significantly lower density of CD163+ cells infiltrating tumors on the *Ndst1*f/f *CD11cCre*+ mutant background (Fig. 3A, right; *P = 0.02 for difference in means). (B) Representative photomicrographs of macrophage (arrows) distribution under IHC staining in control mice are presented to demonstrate: F4/80+ cells in non-tumor lung parenchyma (left panel, Bar=100μm) or tumor-associated F4/80 cells (middle panel, Bar=100μm); while CD163+ cells showed distinct tumor-association/infiltration (right panel Bar=100μm). A separate high-magnification view is shown within full thickness of an adenomatous lesion (Bar=20μm).

*Macrophage profile supports an early cellular anti-tumor phenotype in Ndst1f/f CD11cCre+ mutants:* In the Kras spontaneous lung tumor model, we also profiled using IHC lung tissue labeling with a broad marker for tissue macrophages (F4/80); and with IHC using an antibody to CD163, a marker for M2-type tumor-associated macrophages. The latter have been associated with promotion of tumor growth and poor survival in carcinomas [[8], [9], [10]]. The presence of macrophages staining with F4/80 on the background lung tissue of inducible Kras-transgenic *Ndst1*f/f *CD11cCre*+ mutant mice and *Cre-* controls was quantitatively comparable among genotypes. Staining with antibody to CD163 revealed an infiltrative pattern associated with the early adenomatous lesions (Fig. 3B, right photomicrographs). Upon quantifying infiltrated cells per high power field, while the difference in mean tumor-associated F4/80+ cells between genotypes did not meet statistical significance, there was significant reduction in the density of tumor-associated CD163+ infiltrating cells among lung tumors in *Ndst1*f/f *CD11cCre*+ mutant mice (*P* = 0.02 for difference in means; Fig. 3A, right).

## Discussion

We recently demonstrated that antigen presenting cells carrying a CD11c driven mutation in the major cell-surface glycan sulfating enzyme Ndst1 are associated with augmented induction of anti-tumor CD8+ cytotoxic T cells relative to that of wildtype APCs in model lewis lung carcinoma tumors [1]. In this study, we sought to examine how this host myeloid cell driven mutation might affect early spontaneous tumor formation in the lung using a Kras mutation driven inducible model that initiates with airway-centric adenomas as pre-cancerous lesions.

When noting that the APC-directed *Ndst1* mutation is associated with a reduced frequency of early tumors visible by microscopy in the lungs of mutant mice (Fig. 1C), we wondered if perhaps the mutation might affect any balance of Treg and effector T cells that may regulate tumor formation in the wildtype state. Unlike our more advanced model of macroscopic Lewis Lung carcinoma tumors (LLCs) on the mutant background, where CD8+ effector T cell infiltration is driven, and where tumor antigen load differences may be more advanced (driving acquired anti-tumor immunity), we did not note up-regulation of CD8+ T cells into the early tumors (Fig. 2A); instead, we found a phenotype in the formation of early tumor micro-nodules (Fig. 1B) and an effect on Treg infiltration (Tregs per unit tumor mass; Fig. 2B). This was also accompanied by a reduction in M2-type CD163+ tumor-associated cells in mutants (Fig. 3). Curiously, one of the early hallmarks of altered immunity in lung cancer is the presence of regulatory/ suppressor T cells (Tregs) [4], noted within both the tumor lesion as well as the blood in patients with early-stage disease; and this is in addition to a program of intense immune suppression within the early tumor, as compared to normal lung immediately adjacent to the tumor [3].

We questioned what mechanism(s) might operate to yield the curious association of an APC HS mutation with reduced early tumor formation in the Kras driven model. Possibly the “state” of Ndst1 deficient APCs (DCs and/or macrophages) in tumors, including cytokines produced, or the maturational state of such cells (e.g., elevated CD80/86, MHCII expression, as noted in [1]) may activate certain signaling pathways that drive Ndst1 deficient APCs away from the typically tolerogenic state induced by the early cancer microenvironment. The latter is associated with Treg induction due to cell-surface molecules and/or the cytokine milieu [11]. Possibly, the genetic strategy we employed may also “tip” a balance of maturation or products expressed by APCs in early lung cancers in a way that resists accelerated tumor growth as a result of inhibited Treg expression or expansion within such tumors. Whether some FOXP3 expression takes place in non- T cells (i.e., inhibitory transcriptional signaling in expanding tumor cells) in this model is unclear. Either way, this transcriptional regulatory pathway typically expressed in Tregs that promotes early tumor formation in control Kras mutant tumor bearing mice appears to be suppressed in the setting of APCs expressing under-sulfated HS. Recent work points also to a key interplay between tumor associated macrophages (TAMs) and tumor Treg cells, where TAM co-localization with Tregs mostly sustains the suppressive tumor micro-environment [12]. This may contribute to “resistant” attempts at anti-PD-1 ICI in clinical lung cancer as a result of Treg expression of PD-1 receptors. In new work by Martinez-Usatorre and others [12], depletion of TAMs was associated with Treg down-regulation (along with FOXP3 down-regulation) as well as restoration of tumor inhibition and regression under PD-1 blockade in the models. This may be relevant also in light of the observations herein, where down-regulation of CD163+ TAMs in *Ndst1*f/f *CD11cCre*+ mutants (Fig. 3) was also associated with a tumor phenotype showing reduction in FOXP3+ cells associated with tumors in mutant mice (Fig. 2B). Harnessing this in the setting of early tumor formation might have translational potential.

In the lung, in addition to Ndst1 deficiency in a relatively broad CD11c+ expressing population of DCs, the expression of CD11c by alveolar and interstitial macrophages would lead to some degree of Ndst1 deficiency in the tumor macrophage population of *Ndst1*f/f *CD11cCre*+ mutants during early tumor growth. Upon further characterizing this subset of cells during early tumor formation using CD163 as a marker for M2-subset macrophages, there was distinct infiltration of lung tumors in Kras transgenic mice (Fig. 3B, right). Further quantification of the tumor-specific density of this subset of cells in *Ndst1*f/f *CD11cCre*+ mutant versus *Cre*- control tumor-bearing mice revealed a significantly lower CD163+ cell density in mutant tumors (Fig. 3A). Interestingly, CD163+ macrophages are associated with reduced tumor effector functions (designated “non-cytotoxic,” in contrast to inflammatory-type M1 macrophages); and in carcinoma studies, the CD163+ M2 subset is known for co-expression or tumor-induction of vascular endothelial growth factor (VEGF) family molecules (which drives early tumor vascularization) [8, 13], immunosuppressive cytokines, and poor outcomes [8−10]. The reduced presence of such cells along with a reduction in the frequency of tumor formation on the *Ndst1*f/f *CD11cCre*+ mutant background (Fig. 1) may point to a unique anti-tumor innate immunologic phenotype by *Ndst1* deficient macrophages. This may also precede and possibly act independent of the anti-tumor potential of *Ndst1* deficient DCs that appear to boost acquired cytotoxic CD8+ T cell responses against growing macroscopic carcinomas [1]. More generally, in early tumors, glycan modification may serve as a novel post-translational approach to harness an anti-tumor TAM functional state. In parallel to this, in the early tumor as CD8+ T cells in the tumor microenvironment begin to respond to specific tumor antigen stimuli, new work has shown that unique TAM transcriptional regulation via novel RNA methylation programs may affect the balance of CD8+ T cell effector versus exhaustion mechanisms [14]. In this way, targeting unique epigenetic pathways that maintain an anti-tumor TAM transcriptome while discovering unique post-translational modifications in TAMs that independently promote anti-tumor potential (e.g., via an APC-targeted glycan targeting approach presented here) provides key insights on strategies with translational and clinical potential to modulate both Treg cells and early CD8+ T cell effector functions in the early lung cancer microenvironment.

Further work is needed to understand how glycan under-sulfation affects innate tumor immune signals induced by targeted myeloid cells, and a particular target of interest may be the anti-tumor functional capacity of TAMs during early tumor formation, as these cells are targeted by the *Ndst1*f/f *CD11cCre* genetic strategy we demonstrated herein. The findings point to new opportunities for translational applications that may induce/ promote anti-tumor cellular immunity in spontaneous lung carcinoma. Current and future studies that discover a growing repertoire of APC (including TAM) functional and regulatory targets, with cell-surface glycans demonstrated here, may drive clinical advances that may independently promote an improved anti-tumor T cell balance in the lung cancer microenvironment.

## Conclusion

Genetic under-sulfation of heparan sulfate on a unique class of myeloid-derived CD11c+ antigen presenting cells results in an innate cellular immune profile characterized by reductions in tumor-infiltrating suppressive FOXP3+ cells and CD163+ M2-type tumor-permissive macrophages during early bronchocentric adenoma formation in a Kras-mutant spontaneous lung tumor model. The finding provides mechanistic insight on how targeting glycan sulfation of antigen presenting cells may inhibit early cellular tumor-promoting pathways involved in spontaneous lung tumor development. This may also have implications in novel host-cell centered anti-tumor therapeutics (or even primary or secondary tumor chemo-prevention strategies).
